# Utility of ETCO_2_ to predict hemorrhagic shock in multiple trauma patients

**DOI:** 10.3906/sag-2103-206

**Published:** 2021-10-23

**Authors:** Gülşen ÖZTÜRK ÖRMECİ, Özlem YİĞİT, Oktay ERAY

**Affiliations:** 1 Department of Emergency Medicine, Antalya Training and Research Hospital, Antalya, Turkey; 2 Department of Emergency Medicine, Faculty of Medicine, Akdeniz University, Antalya, Turkey

**Keywords:** Trauma, ETCO_2_, base excess (BE), hemorrhagic shock, emergency department

## Abstract

**Background/aim:**

For identifying hemorrhagic shock in trauma patients, some objective data are needed. The use of base excess (BE) and lactate values have been originated. In this study, it was aimed to determine the usability of end tidal carbon dioxide (ETCO_2_) in patients with multiple trauma for recognizing hemorrhagic shock.

**Materials and methods:**

Patients who were admitted to the emergency department between June 2019 and February 2020 with high-energy multiple trauma were included in the study. ETCO_2_ and BE values were measured. Correlation coefficients were calculated to determine correlations between ETCO_2_ and BE levels.

**Results:**

One hundred and twenty-two patients were included in the study. Eighty-nine (73%) were men and 33 (27%) were women, and the mean age of the study population was 38.70 ± 19.18. The mortality rate was 14.8% in the study population. The correlation between ETCO_2_ and BE values was significant (r: 0.27) and in the same range in the Bland-Altmann analysis. ETCO_2_ levels above 35 were specific for stage 1 hemorrhagic shock. ETCO2 levels below 30 were sensitive for stage 2 and 3 hemorrhagic shocks and when the levels were measured below 22 it was found specific for stage 4 shock. The specificity increased to 99% at levels below 18. The sensitivity for ETCO_2_ values below 22 for predicting mortality was 33.33%, the specificity was 89.42%, the positive predictive value was 35.29% and the negative predictive value was 88.57%. The sensitivity for BE values below -10 for predicting mortality was 50%, the specificity was 93.27%, the positive predictive value was 56.25% and the negative predictive value was 91.51%.

**Conclusion:**

ETCO_2_ measurement can be a useful parameter as a noninvasive and simple technique in predicting and classifying hemorrhagic shock, which is the leading cause of mortality in trauma patients. Mortality rates increased when ETCO_2_ was measured below 22 and these patients are more likely to be in the critical hemorrhagic shock state.

## 1. Introduction

Hemorrhage is the predominant cause of preventable deaths after traumatic injury [1]. Identifying, quickly controlling hemorrhage, and initiating resuscitation are therefore crucial steps in assessing and managing such patients. For identifying hemorrhagic shock, classification systems based on vital parameter changes are used. However, some objective data are needed for identifying patients with early-stage hemorrhagic shock in which vital signs are normalized with compensatory mechanisms and abnormalities cannot be detected [2]. For this purpose, the use of base excess (BE) and lactate values have been originated. In light of the positive results obtained from these studies, BE which is one of the parameters of tissue hypoperfusion, took its place in shock parameters [2]. The relation of BE value with blood and blood product requirement, morbidity, and mortality has been shown [3]. Furthermore, end tidal carbon dioxide (ETCO_2_) measurements have been researched on the previous studies whether the levels could be a guide in early shock parameters or not. It was found that the ETCO_2_ values were compatible with blood gas parameters [4,5,6,7].

The amount of carbon dioxide (CO_2_) and in relation with this, measured in breathing air ETCO_2_; is determined by alveolar ventilation, pulmonary perfusion, and ventilation-perfusion defect [2]. For this reason, the ETCO2 value is expected to change in cases such as hemorrhagic shock that causes low blood flow. Since the ETCO_2_ value can be easily measured noninvasively in patients with or without intubation, using these values as a hemorrhagic shock classification parameter can be a very good guide in patient management. In this study, it was aimed to determine the usability of ETCO_2_ in patients with multiple trauma for recognizing hemorrhagic shock. The primary goal was to determine whether ETCO_2_ is a usable parameter as BE values in arterial blood gas for hemorrhagic shock classification. The secondary purpose was identifying ETCO_2_ threshold values that can be used to predict the need for intensive care unit admission and mortality.

## 2. Materials and methods

This prospective methodologic study was conducted in a tertiary care university hospital emergency department between June 2019 and February 2020, with an Institutional Ethics Committee approval (08.05.2019/433) in accordance with the Declaration of Helsinki. The study was funded by using the short-term grant (1-year duration) provided by the University Research Foundation (grant no. TTU-2019-4924).

### 2.1. Inclusion criteria of patients

Patients aged 18 years and over, who were brought to the emergency department by the prehospital ambulance team and considered to have serious life-threatening injuries or evidence of high-energy impact according to the ATLS (Advanced Trauma Life Support) guidelines (8) were reevaluated for study inclusion. The emergency medicine specialist and/or senior research assistant doctor did the reevaluation. Patients injured with a high energy impact mechanism and showing the evidence of clinically suspicious life-threatening injuries and with ‘multiple trauma’ were included in the study. A diagnosis of “multiple trauma” was defined as the presence of two or more separate injuries of body systems (head, thorax, abdomen, pelvis, long bones, spine), at least one or a combination of which endangers the patient’s life.

### 2.2. Exclusion criteria of patients

1. Patients who were referred to our hospital from another medical center and have been given resuscitative treatments for hemorrhagic shock in the center before referral
2. Pregnant patients and children

ETCO_2_ values were measured by capnography in patients who were considered to have high-energy trauma before applying any sedative agent and resuscitative therapy for hemorrhagic shock. Massimo Emma Mainstream Emergency Portable Real-Time capnography device (EMMA™ Capnograph, USA) which was purchased within the scope of University Research Foundation support was used for ETCO_2_ level measurement in the patient.

All physicians and paramedics working in airway management in the emergency department were informed about the usage of the device, before starting the study. An airway mask attached to the capnography device was used to measure ETCO2 levels in patients with regular spontaneous breathing and without intubation indication immediately in the early period. In these conscious patients with spontaneous regular breathing, the ETCO_2_ value after 6 breaths given to the face mask was recorded.

In patients with irregular breathing or confusion and with any other early intubation indication, the ETCO_2_ value was recorded after giving 6 breaths with a balloon valve mask attached to the capnography device. During these measurements, patients’ standard advanced trauma life support applications were not interrupted, and no changes have been made to the management process. Arterial blood samples were taken from all patients in accordance with the recommendation of using the BE value in hemorrhagic shock classification in the latest ATLS guideline. Both radial and femoral regions were used for sampling. Blood gas samples taken from the patients were studied on the calibrated Siemens RAPIDLab 1265 device (Siemens Healthineers, Germany). The measured BE values were used for comparisons.

The study form was filled by the emergency physician for all patients included in the study. In the study form; demographic data and underlying chronic diseases of patients, injured systems of the body, the vital parameters used for hemorrhagic shock classification (blood pressure, pulse rate, respiratory rate, O2 saturation, urine output, need for blood products, fluids, vasopressors and transamine), BE levels measured in arterial blood sample, measured ETCO_2_ values, hospitalization status (inpatient clinic, intensive care unit (ICU) or referral to another hospital), length of stay in the intensive care unit, the total length of stay in the hospital and mortality presence were recorded. Injury Severity Score (ISS) and Revised Trauma Score (RTS) were also calculated for all patients and recorded in the study form.

### 2.3. Statistical analysis

Demographic data are presented in raw numbers and percentages. All continuous data with a normal distribution were presented as means ± standard deviation (SD), whereas, nonnormal data were presented as the median and interquartile range (IQR). Chi-square tests were used for the comparison of categorical variables between the groups. A Shapiro-Wilk test was used to determine the distribution of the numerical variables; nonnormal data were evaluated using the Mann-Whitney-U test. Spearman’s correlation coefficients were calculated to determine correlations between ETCO_2_ and BE levels and other variables. The main outcome of ETCO_2_ and BE agreement was determined using Bland-Altman analysis.

The best cutoff point was identified and reported in terms of sensitivity, specificity and receiver operator characteristic curves (ROC) were generated to analyze the optimal cut-off level of ETCO_2_ and BE. The accuracy was considered with AUC along with its standard deviation (SD) or 95% confidence interval (95% CI). The sensitivity, specificity, positive and negative likelihood ratio (PLR and NLR) values were calculated for detecting the value of the optimal cut-off level of ETCO_2_ and BE in determining mortality. Logistic regression analysis with 95% confidence intervals (CIs) was performed to report odds ratios (OR) of the factors predicting mortality in the first 24 h. All statistical analyses were performed using IBM SPSS statistics data editor version 25.0 (Chicago, IL, USA). A p value of < 0.05 was considered statistically significant.

## 3. Results

A total of 180 patients with suspected high energy mechanisms and/or serious injuries were brought to the emergency room by the ambulance team. Fifty-eight of them were excluded from the study for the lack of serious life-threatening multiple injuries by two independent emergency medicine specialists. The final analysis was done with the remaining 122 patients. Of the total 122 patients who participated in the study, 89 (73%) were men and 33 (27%) were women, and the mean age of the study population was 38.70 ± 19.18 years (min 18–max 82, median 32.5). Mortality was significantly high for patients with head-neck trauma (83.3% of all mortalities, p: 0.02), for patients with underlying diabetes (22.2% of all mortalities, p: 0.03) and hypertension (44.4% of all mortalities, p: 0.00). Patients with thorax trauma needed more ICU admission than other trauma types (79.8% of all ICU admissions, p: 0.05), however the mortality rate was insignificant (88.9% of all mortalities, p: 0.17). The other parameters were not statistically significant for ICU admission. The demographics of the study patients are given in Table 1. The vital parameters and trauma severity scores were shown in Table 2.

The sensitivity for ETCO_2_ values below 22 for predicting mortality was 33.33% (95% CI: 13.34% to 59.01%), the specificity was 89.42% (95% CI: 81.86% to 94.60%), the positive predictive value was 35.29% (95% CI: 18.76% to 56.31%) and the negative predictive value was 88.57% (95% CI:84.74% to 91.54%). The sensitivity for BE values below -10 for predicting mortality was 50 % (95% CI: 26.02% to 73.98%), the specificity was 93.27% (95% CI: 86.62% to 97.25%), the positive predictive value was 56.25% (95% CI: 35.43% to 75.08%) and the negative predictive value was 91.51% (95% CI:87.13% to 94.49%).

Intensive care unit stay of patients was 8.14 days on average, minimum of one day to maximum of 80 days. The mean value of the total length of stay in the hospital of patients was found 15.2 days. Resuscitative procedures applied to the patients were shown in Table 3, and emergency department outcomes were shown in Table 4. The analysis of the patients who died were given in Table 5. Spearman’s correlation test was applied to evaluate the consistency between ETCO_2_ and the need for blood products of patients and a significant correlation was found between the two tests (correlation coefficient r: 0.29). The decision of the blood product need was considered according to the vital signs of the patients.

### 3.1. Comparison of BE and ETCO_2_ values

The median of measured BE in the arterial blood gas sample was found to be -4 (min. -26, max. 6, IQR -7.2 and - 2). There were 16 (13%) patients with BE values -10 and less, and 33 (26.2%) patients’ value was detected between -6 and -10.

**Figure 1:**
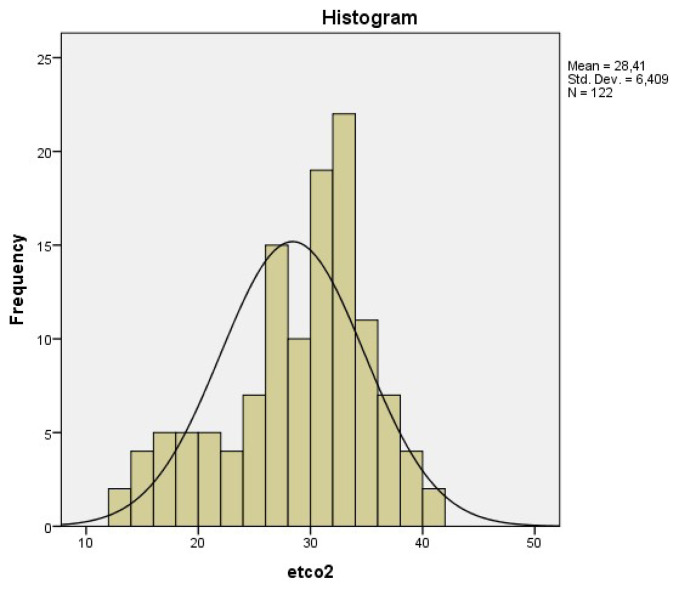
The distribution of ETCO2 levels of study patients.

The mean of ETCO_2_ values was 28.4 ± 6.4 mmHg (Median: 30, min. 13, max. 41, IQR 24–33). ETCO_2_ values were measured in normal (35–45) ranges for only 18 (14.6%) patients. The distribution of ETCO2 levels of study patients was shown in Figure 1.

Spearman’s correlation test was applied to evaluate the consistency between ETCO_2_ and BE values of patients and a significant correlation was found between the two tests. (correlation coefficient r: 0.27).

**Figure 2:**
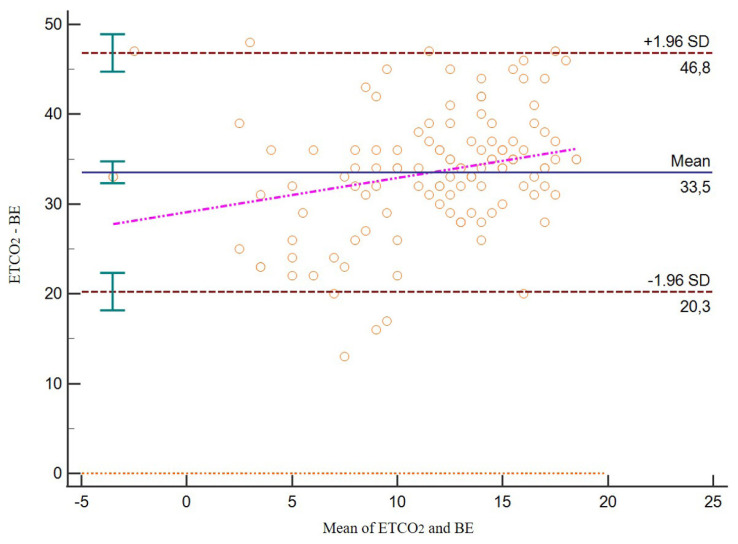
Bland-Altman analysis for ETCO_2_ and BE.

ETCO_2_ and BE values were found to be in the same range in the Bland-Altmann analysis (Figure 2).

### 3.2. Determination of ETCO_2_ threshold values for hemorrhagic shock classification

**Figure 3:**
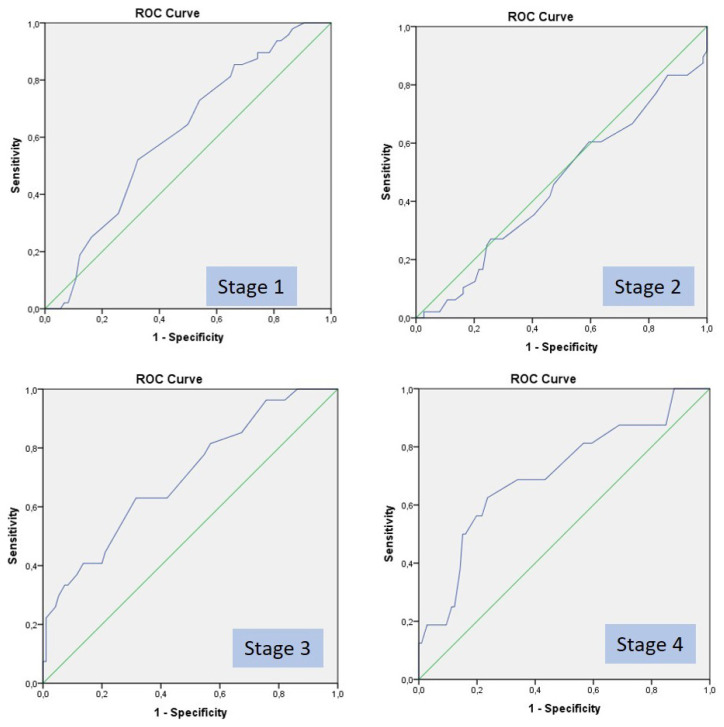
ROC curves for hemorrhagic shock stages and ETCO_2_ levels.

The correlation between ETCO2 values and shock stages which were determined by classical shock parameters were analyzed, and the sensitivity and specificities of the ETCO_2_ values that can be used to determine the stages were calculated. The number of patients in all stages and the sensitivity and specificity of ETCO2 values below 22, and AUC are shown in Table 6. The ROC curves of all stages are shown in Figure 3.

Patients with normal vital parameters were classified as Stage 1. Normal vital parameters were defined as heart rate <100/min, systolic blood pressure >90 mmHg, respiratory rate within 14–20/min. ETCO_2_ levels above 35, which is the lower limit of normal range, the sensitivity was 10%; however, the specificity was 99%. When the threshold for ETCO_2_ levels decreased to 28, the sensitivity was 65% and the specificity was 50%. When the threshold for ETCO_2_ levels decreased to 22, the sensitivity was 93% and the specificity was 19%.

Patients with tachycardia (heart rate ≥ 100) were classified as Stage 2. The sensitivity of ETCO2 levels below 30 was found 46%, and the specificity was 53%. The specificity increased to 80% at levels below 22. When the pulse rate threshold was 86, the sensitivity increased to 82%.

Patients with hypotension (systolic blood pressure ≤ 90 mmHg) were classified as Stage 3. The sensitivity of ETCO_2_ values below 30 was found 77%, and the specificity was 56%. The specificity increased to 87% when ETCO_2_ value was 22 and below, and 99% when ETCO2 value was 17 and below.

Patients with a BE value -10 and less were classified as Stage 4. The sensitivity of ETCO_2_ levels below 27 was found 68%, and the specificity was 76%. The specificity increased to 86% at levels below 22, and 99% at levels below 18.

### 3.3. Subgroup analysis of patient with mortality

Mann Whitney U test was used to determine the worthiness of ETCO_2_ and BE values in predicting mortality since the data did not follow the normal distribution. Mann-Whitney U test revealed that ETCO_2_ values were significantly lower in the mortality group (Md = 27, n = 18) compared to the group without mortality (Md = 30, n = 104), U = 630.5, z = −2.21, p = 0.027. BE values were also significantly lower in the mortality group (Md = −4, n = 18) than survivors (Md = −9, n = 104), U = 520, z = −3.01, p = 0.03.

**Figure 4:**
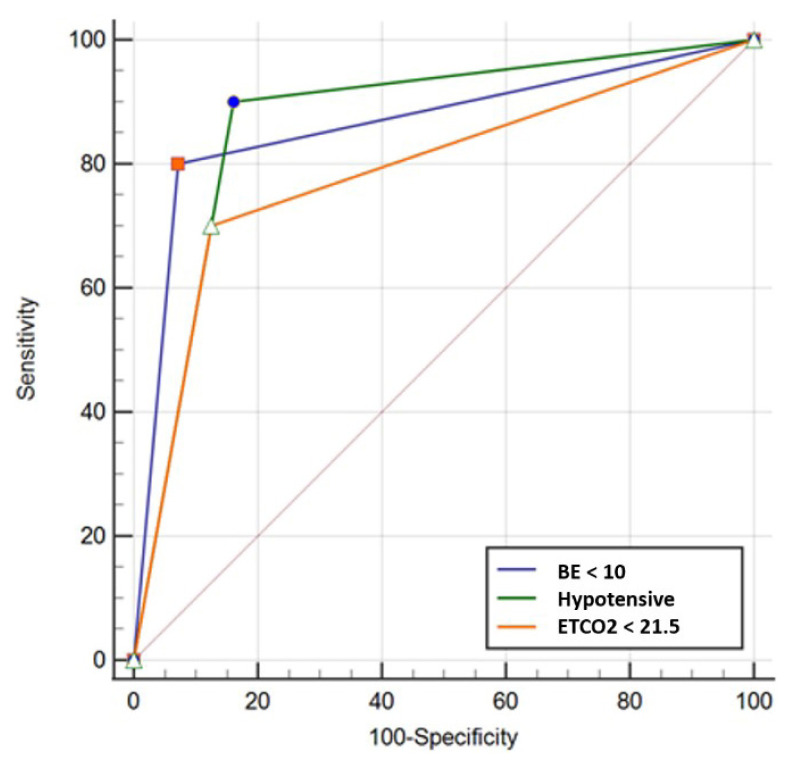
ROC curve comparisons of ETCO2, BE, and hypotension for predicting mortality in the first 24 h.

In the ROC curve analysis for determining the mortality in 24 h, the sensitivity of ETCO_2_ levels below 22 were found 70%, and the specificity was 88%. The sensitivity increased to 94% for ETCO_2_ levels below 17. For predicting mortality in the first 24 h ETCO_2_ AUC was 0.748 (CI = 0.551–0.946). For predicting overall mortality, the sensitivity of ETCO2 levels below 22 was found 44%, and the specificity was 90%. ETCO_2_ AUC was 0.663 (CI = 0.515–0.811). The ROC curve comparisons of three parameters were shown in Figure 4.

The odds of ETCO_2_ levels below 22 were 16.33 (95% CI = 3.77–70.60; p: 0.00) for mortality in the first 24 h. A logistic regression analysis to investigate the factors affecting mortality was conducted. ETCO_2_ levels below 22, the presence of hypotension and BE levels below -10 were found statistically significant for predicting mortality in the first 24 h in the model (Table 7).

## 4. Discussion

In our study, we evaluated the usability of the noninvasively measured ETCO_2_ levels and the compatibility of these values with BE values measured in the patients’ blood gas for predicting the presence of hemorrhagic shock in patients brought to the emergency department after high energy trauma. It was found that the ETCO_2_ values showed a significant correlation with BE levels, and could significantly predict the mortality development in the early period of trauma care. We suggest that the threshold values determined for ETCO_2_ could be used more than 90% sensitivity and specificity both classifying hemorrhagic shock and predicting admission to the intensive care unit and mortality.

The correlation between the ETCO_2_ levels and the parameters such as lactate, bicarbonate, base deficit, carbon dioxide levels measured in blood gas samples was investigated in literature in previous years [4,5,6,7]. While the first studies on this subject were mostly experimental animal studies, there have been studies conducted in recent years on patients with various clinical presentations or admitted to the emergency department or hospitalized in the intensive care unit. In animal studies, a statistically linear correlation was found between ETCO_2_ which measured from the endotracheal tube and partial CO2 in the blood gas [9]. In another study, it was stated that tissue oxygen saturation and ETCO_2_ values are good indicators of acute-phase shock [10].

Williams et al. measured the ETCO_2_ value with a nasal cannula in trauma patients and found the mean ETCO_2_ value of all patients as 32.2 mmHg [11]. The majority of 171 patients included were men and their mean age was 43 years, and these were similar to our study results. In our study, the mean ETCO_2_ value was found at 28.4 and the most common system injury in patients was thoracic trauma. This may be the reason for the lower mean values. ETCO_2_ value was not statistically significant in determining the need for blood products in the study mentioned above. However, we found a significant correlation in the present study.

Using only classical shock parameters may not be sufficient for predicting highly intensive care unit need and mortality in the early period of patient care. A recent retrospective review including 630,635 trauma patients concluded that almost half of trauma patients do not meet the criteria for any ATLS shock class and uncategorized patients had higher mortality [12]. The ATLS classification system does not help identify many patients in severe shock and we need more objective measurable parameters. We know that the compensatory response to hemorrhagic shock is variable in different cohorts of patients, that is, the elderly, athletes, and in pregnancy. Also, confounders such as the presence of traumatic brain injury or spinal cord injury, medication or illicit drug use, and the medical history affect hemodynamics [13]. In trauma patients, there is an interrelationship between derangements of vital parameters but not to the same degree as that suggested by the ATLS classification of shock [14,15]. It was considered that other measures, such as base deficit or serum lactate level may prove useful in predicting the severity of shock and distinguishing major from minor injury in trauma patients [16]. BE which is one of the parameters of tissue hypoperfusion, took its place in shock parameters [8]. However, we all know that arterial puncture for sampling arterial blood gas is an invasive, painful, and relatively difficult procedure, also can cause some local complications. Wrong measurements can be detected because of technical faults such as not expressing the heparin from the preheparinized syringe before taking the sample or not optimizing the timing of the measurement. Sometimes arterial blood samples were not able to be taken before fluid administration or other resuscitative procedures given to the patients who were unstable and this could change the results of the test. ETCO_2_ measured by a noninvasive method rises out as a usable method. When we look at the threshold values obtained by ROC analysis, low ETCO_2_ values are compatible with shock classifications determined according to classical vital parameters and BE values in blood gas and it has been found to be significant in predicting intensive care unit admission, and also mortality. Especially, ETCO_2_ values measured at 22 and below are important in detecting critical hemorrhagic shock patients according to both vital parameters, sensitivity, and specificity values exceeding 90% in predicting the need for intensive care unit hospitalization. Also, it is seen that the median of ETCO_2_ values of patients who died in the first 24 h is 20. These values are very similar to the 18 values found for patients who died in the Childress et al. study [17]. It may be appropriate to confirm these threshold values with studies with more patients.

When using classical shock parameters, patients with normal vital signs were classified as Stage 1. Predicting this early phase in a patient is only a suspicion, and BE values also did not add clear information for this phase. The values above 35, which is the lower limit of the normal range, the sensitivity was very low and the specificity was higher as it could be predicted. With the decrease in ETCO_2_ values, the sensitivity tended to increase. We found that ETCO_2_ levels below 28 indicated a sensitivity of 65%, and when the threshold for ETCO_2_ levels decreased to 22, the sensitivity was 93% for stage 1. This means that if you see the lower levels of ETCO_2_ rather than normal values, it could be predictive for a hemorrhagic shock even though normal vital parameters. We can mention that ETCO_2_ values under 28 which were our mean value for ETCO_2_ could be a warning sign for an early phase of shock, and if the values are under 22 it could be a very early indicator for stage 1 hemorrhagic shock in patients with normal vital signs. The threshold values which can predict stage 1- the very early phase of hemorrhagic shock can be detected by further new studies. Only 14.6% of patients have ETCO_2_ values in the normal range in our study.

### 4.1. Limitations of the Study

Our study has some limitations. Due to the small number of patients, the reliability of the ETCO_2_ threshold values may be limited. Especially in patients who are not intubated, the measurement of ETCO_2_ value with the capnography device and breathing compliance may be limited when they first come to the emergency room.

## 5. Conclusion

In this study, it has been shown that ETCO_2_ measurement can be a useful parameter as a noninvasive and simple technique in predicting and classifying hemorrhagic shock, which is the leading cause of mortality in trauma patients. We found that the mortality rates of the patients increased when ETCO_2_ is measured below 22 mmHg and it is possible to say that patients are more likely to be in the critical hemorrhagic shock state.

## Figures and Tables

**Table 1:** Patient’s demographics.

Age (years) (mean ± SD)	38.52 ± 19.25; Median 31.5 (min 18–max 82)				
Sex	n (%)				
Male	89 (73)				
Female	33 (27)				
			*Mortality (18 total deaths)		
Types of trauma	n (%)	ǂ ICU admission (%)	n /%	χ2 value	p value
Head-neck trauma	72 (59)	58 (80.5)	15 (83.3)	5.162	0.02
Thorax trauma	93 (76.2)	79 (84.9)	16 (88.9)	1.868	0.17
Abdomen trauma	53 (43.4)	45 (84.9)	11 (61.1)	2.683	0.10
Pelvic trauma	32 (26.2)	23 (71.9)	6 (33.3)	0.551	0.45
Limb trauma	75 (61.5 )	62 (82.7)	10 (55.6)	0.312	0.57
			*Mortality (18 total deaths)		
Comorbidities	n (%)	ǂICU admission (%)	n /%	χ2 value	p value
Diabetes mellitus	11 (9)	9 (81.8)	4 (22.2)	4.489	0.03
Hypertension	18 (14.8)	12 (66.7)	8 (44.4)	14.599	0.00
CAD	6 (4.9)	4 (66.7)	2 (11.1)	1.732	0.18
COPD	1 (0.8)	0 (0)	1 (100)	5.826	0.01

ICU: Intensive care unit, CAD: Coronary artery disease, COPD: Chronic obstructive pulmonary disease

ǂ ICU admission (%) represents the number and percentage of patients hospitalized to the intensive care unit with that type of trauma and comorbidity. The patient with COPD died in the emergency room, for this reason, he did not hospitalized in the ICU.

* Mortality (n/%): There are 18 total deaths in the study population. n/% represents the number and percentage of patients within these 18 deaths in the study group with that type of trauma and comorbidity.

**Table 2:** Vital parameters and functional status of patients at the time of admission.

	Number of patients (%)
Blood pressure (mmHg)	
No hypotension	95 (77.9%)
Hypotension (systolic blood pressure ≤ 90 mmHg)	27 (22.1%)
• Systolic blood pressure mean value (all study patients)	• 110.29 ± 24.15 mmHg
• Diastolic blood pressure mean value (all study patients)	• 67.64 ± 14.49 mmHg
Heart rate/minute	
No tachycardia	52 (42.6%)
Tachycardia (pulse ≥ 100 beats/min)	70 (57.4%)
• Heart rate mean value (all study patients)	• 100.89 ± 20.56 beats/min (min 57– max 182)
Respiratory rate /min	
No tachypnea	62 (50.9%)
Tachypnea (respiratory rate > 20 /min)	60 (49.1%)
• Respiratory rate mean value (all study patients)	• 18/min (min 12– max 32)
Glasgow coma score (GCS)	
GCS 15	72 ( 59%)
GCS 12 – 14	18 (14.8%)
GCS 9 – 11	6 (5%)
GCS 8 and less	26 (21.3%)
Revised trauma score (RTS)	
RTS 8 (good prognosis)	79 (64.8%)
RTS < 4	10 (8.2%)
• RTS median value	• 8 (min 2, max 8)
Injury severity score (ISS)	
ISS > 16 (poor prognosis)	99 (81.1%)
ISS < 16 (good prognosis)	23 (18.9%)
• ISS median value	• 22 (min 2, max 66)

SD: Standard deviation

**Table 3:** Resuscitative procedures applied to patients.

Treatment applied	n (%)
Bolus fluid infusion	107 (86.5)
Blood product infusion	39 (32)
Massive blood transfusion	4 (3.3)
Vasopressor medications	19 (15.6)
Tranexamic Acid	39 (32)

**Table 4:** Emergency department outcomes of patients.

Emergency clinic outcome	n (%)
ICU hospitalization	94 (77)
Inpatient clinic hospitalization	19 (15.6)
Referral to another hospital (for ICU)	5 (4.1)
Death in the emergency room	4 (3.3)
Total number of deaths	18 (14.8)
• First 24 h (including who died in the emergency department)	10 (8.2)
• First 7 days	4 (3.3)
• First 1 month (after 7 days)	1 (0.8)
• First 3 months (after 1 month)	3 (2.5)

ICU: Intensive Care Unit

**Table 5:** Analysis of the patients who died.

	n (%)
Total mortality	18 (14.8)/122 (total patients)
• First 24 h	• 10 (8.2%)/18
Age (years) (Mean ± SD)	51.06 ± 5.78
Sex (male %)	11 (61.1%)/18
ETCO_2_ (median, IQR) for all deaths	27, IQR [17–30] (min 13–max 38)
• ETCO_2_ (median, IQR) for deaths occurred in the first 24 h	20, IQR [14–30] (min 13–max 35)
ETCO_2_ < 22	8 (38.1)/18
• ETCO2< 22 (for deaths occurred in the first 24 h)	• 7 (70)/10
BE (median, IQR)	-9, IQR [-14.75_-3.75] (min −26–max 1)
BE < −10	9 (50)/18
• BE < −10 (for deaths occurred in the first 24 h)	• 8 (80)/10
Hypotension	12 (66.7)/18
Tachycardia	13 (72.2)/18
RTS < 4	8 (44.4)/18
ISS > 16	18 (100)/18
Transfusion	15 (83.3)/18
Bolus fluid resuscitation	17 (94.4)/18
Vasopressor	10 (55.6)/18

SD: Standard deviation, IQR: Interquartile ratio, ETCO2: End tidal carbon dioxide, BE: Base Excess, RTS: Revised Trauma Score, ISS: Injury Severity Score

**Table 6:** The number of patients in all stages and the sensitivity and specificity of values below ETCO2 < 22 for these stages.

	Patient n (%)	Sensitivity (%)	Specificity (%)	AUC
Stage 1	48 (39.3%)	93	19	AUC = 0.611, CI = 0.511–0.711
Stage 2	48 (39.3%)	16	80	AUC = 0.456, CI = 0.350–0.562
Stage 3*	26 (22.1%)	40	87	AUC = 0.703, CI = 0.590–0.815
Stage 4*	16 (13.1%)	50	85	AUC = 0.704, CI = 0.558–0.849

* Stage 4 patients were classified with BE values and stage 3 patients were classified with hypotension. For this reason, some patients in these two groups were overlapped, so the total patient number seems to be higher than the study total number.

**Table 7:** Logistic regression analysis for factors predicting mortality in the first 24 h.

	Odds ratio	B	S.E.	(95% Confidence Interval)		p value
				Lower	Upper	
ETCO2 below 22	16.33	3.217	1.331	1.83	338.79	0.016
BE below −10	52	3.779	1.324	3.26	586.25	0.004
Hypotension	47	2.859	1.349	1.24	245.24	0.034
Tachycardia	3.22	-0.759	1.531	0.65	15.87	0.131

ETCO2: End tidal carbon dioxide, BE: Base Excess
